# Assessment of acute bone loading in humans using [^18^F]NaF PET/MRI

**DOI:** 10.1007/s00259-019-04424-2

**Published:** 2019-08-05

**Authors:** Bryan Haddock, Audrey P. Fan, Scott D. Uhlrich, Niklas R. Jørgensen, Charlotte Suetta, Garry Evan Gold, Feliks Kogan

**Affiliations:** 1 Department of Clinical Physiology, Nuclear Medicine and PET, Rigshospitalet, Copenhagen University Hospital, Valdemar Hansens Vej 3-13, 2600 Glostrup, Denmark; 2 Department of Radiology, Stanford University, Stanford, CA USA; 3 Department of Mechanical Engineering, Stanford University, Stanford, CA USA; 4 Department of Clinical Biochemistry, Rigshospitalet, Copenhagen University Hospital, København, Denmark; 5 OPEN, Odense Patient data Explorative Network, Odense University Hospital/Institute of Clinical Research, University of Southern Denmark, Odense, Denmark; 6 Geriatric Research Unit, Bispebjerg-Frederiksberg and Herlev-Gentofte Hospitals, Copenhagen University Hospital, København, Denmark; 7 Department of Bioengineering, Stanford University, Stanford, CA USA; 8 Department of Orthopaedic Surgery, Stanford University, Stanford, CA USA

**Keywords:** PET/MRI, Hybrid imaging, Knee, NaF, Kinetics, Bone, Perfusion

## Abstract

**Purpose:**

The acute effect of loading on bone tissue and physiology can offer important information with regard to joint function in diseases such as osteoarthritis. Imaging studies using [^18^F]-sodium fluoride ([^18^F]NaF) have found changes in tracer kinetics in animals after subjecting bones to strain, indicating an acute physiological response. The aim of this study is to measure acute changes in NaF uptake in human bone due to exercise-induced loading.

**Methods:**

Twelve healthy subjects underwent two consecutive 50-min [^18^F]NaF PET/MRI examinations of the knees, one baseline followed by one post-exercise scan. Quantification of tracer kinetics was performed using an image-derived input function from the popliteal artery. For both scans, kinetic parameters of K_i_^NLR^, K_1_, k_2_, k_3_, and blood volume were mapped parametrically using nonlinear regression with the Hawkins model. The kinetic parameters along with mean SUV and SUV_max_ were compared between the pre- and post-exercise examinations. Differences in response to exercise were analysed between bone tissue types (subchondral, cortical, and trabecular bone) and between regional subsections of knee subchondral bone.

**Results:**

Exercise induced a significant (*p* < <0.001) increase in [^18^F]NaF uptake in all bone tissues in both knees, with mean SUV increases ranging from 47% in trabecular bone tissue to 131% in subchondral bone tissue. Kinetic parameters involving vascularization (K_1_ and blood volume) increased, whereas the NaF extraction fraction [k_3_/(k_2_ + k_3_)] was reduced.

**Conclusions:**

Bone loading induces an acute response in bone physiology as quantified by [^18^F]NaF PET kinetics. Dynamic imaging after bone loading using [^18^F]NaF PET is a promising diagnostic tool in bone physiology and imaging of biomechanics.

**Electronic supplementary material:**

The online version of this article (10.1007/s00259-019-04424-2) contains supplementary material, which is available to authorized users.

## Introduction

Acute loading of bone tissue is thought to stimulate bone formation and is of growing interest clinically and in the study of bone physiology. Abnormal bone physiology is not only a key element in joint disease and osteoporosis, but skeletal fragility is directly related to mortality [1–3] and risk of fracture [4]. Studies of bone adaptation to loading have shown that stress on bone cells and strain-mediated fluid flow are crucial in regulating bone metabolism [5–11]. However, the acute response of loading in bone is still poorly understood and difficult to measure in humans in vivo.

Molecular information from PET has shown promise in early detection of metabolic abnormalities of bone metabolism in osteoarthritis [12, 13], associations to bone pain [12–16], and early indication of bone degradation in diseases such as osteoarthritis [14] and osteoporosis [17]. PET has also been used to demonstrate changes in glucose uptake and blood flow in bone marrow in response to exercise loading [18–20]. [^18^F]-sodium fluoride ([^18^F]-NaF) is a well-established bone-seeking agent which may serve as a marker to study bone turnover. In particular, kinetic modeling of dynamic [^18^F]NaF uptake can quantify bone physiology including bone perfusion (K_1_), bone mineralization (k_3_), and tracer plasma clearance (K_i_). Animal studies [8, 21–24] have shown both an acute hyperemia [22, 24] and a large increase in [^18^F]-NaF standard uptake values (SUV) in response to acute loading, which lasts up to 7 days after loading [21, 22, 24]. Furthermore, this response has been positively correlated with the force intensity of the applied load on the bone [21]. The dynamics of [^18^F]NaF uptake after acute bone loading have not been reported in humans. To date, human studies of bone adaptation to loading have been based on changes in structure and mineral density over periods of weeks and months of high-impact exercise. The aim of this study is to evaluate quantitative measures of [^18^F]NaF uptake and tracer kinetics to assess the acute physiological vascular and metabolic response of bone to loading in the human knee using PET/MRI.

## Methods

### Subject population

The study was approved by the Stanford University Institutional Review Board (Stanford University, Administrative Panels for the Protection of Human Subjects). All subjects were informed about the nature of the study and provided written consent prior to participating. The knee joints of 12 healthy subjects (seven females, five males; age: 34 ± 7 years; body-mass index: 23.1 ± 3.3 kg/m^2^) were scanned using [^18^F]NaF PET/MRI before and after performing one-legged step-up and drop–land exercises.

### Exercise protocol

**Fig. 1:**
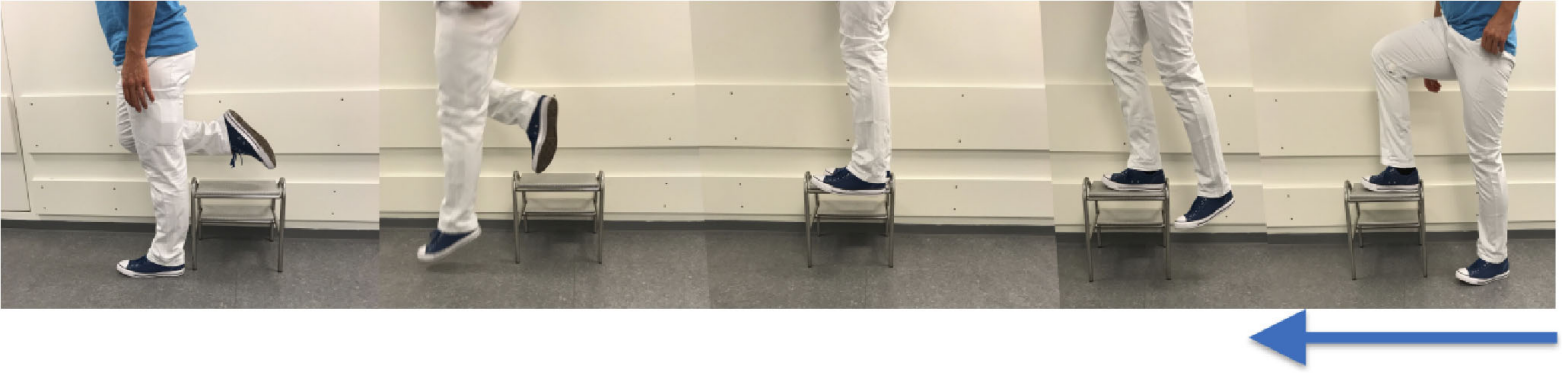
Exercise protocol. Subjects performed 100 repetitions involving stepping up on a 25 cm stool with their right leg and jumping down and landing on their left leg.

After a baseline PET/MRI scan, subjects performed an exercise protocol consisting of 100 repetitions of stepping up on a 25-cm stool using the right leg (step-up), followed by a straight-legged drop jump landing on the left leg (drop–land) (Fig. 1) at a rate of approximately 15 steps a minute. The drop–land exercise induces a high bone-strain magnitude and strain rate, and effectively strengthens bone in humans via a bone adaptation response [25]. The PET/MRI scan was repeated immediately after exercise.

### Bilateral PET/MRI imaging

Simultaneous PET and MRI scanning was performed at baseline prior to exercise and post-exercise. Scanning was performed on a 3 T whole-body time-of-flight PET/MRI hybrid system (GE Healthcare, Milwaukee, WI, USA) with a 16-channel flexible phased-array wrap coil (NeoCoil, Pewaukee, WI, USA) around each knee [26]. Both knees were scanned in one PET bed (field of view = 26 cm) in list mode for 50 min immediately after hand injection of 93 ± 2 MBq of [^18^F]NaF. After exercise, prior to the second injection, 3 min of additional PET scan time was acquired to estimate activity remaining from the first injection. This residual NaF activity was then subtracted from the images used for post-exercise time activity curves and SUV images.

MRI data of both knees was acquired simultaneously with PET data, and included sequences for MR-based attenuation correction (MRAC) and magnetic resonance angiography (MRA). MRA data was acquired using a 3D GRE sequence with imaging parameters: TR/TE = 21/2.1 ms, slices = 18, slice thickness = 1.2 mm, and flip angle = 15°. Data for MRAC [27] were acquired using a 2-point Dixon fat-water, T1-weighted fast spoiled gradient echo sequence with acquisition parameters: TR/TE1/TE2 = 4.1/1.1/2.2 ms; FOV = 50 × 37.5 cm; matrix = 256 × 128; slice thickness/overlap = 5.2/2.6 mm; 120 images/slab; scan time = 18 s.

### Kinetic modelling

Dynamic PET frames were reconstructed to derive the image-derived input function (IDIF) and time–activity curves as previously described [28]. Frame times were 40 × 1 s, 13 × 10 s and 23 × 2 min for the IDIF and 8 × 2 s, 24 × 2 min for r the time–activity curves. Reconstructions were performed using time-of-flight OSEM with three iterations and 21 subsets including corrections for attenuation, scatter, randoms, and dead-time.

The IDIF was derived from [^18^F]NaF activity (kBq/ml) within the popliteal arteries of the knees on the PET/MRI as previously described [28]. Voxels with the highest 10% of NaF activity within the arteries, as determined by the MRA, were included in IDIF analysis. To compare with the IDIF at steady-state, a 3-ml venous blood sample was taken 50 min after the first injection and measured in a well counter. At this point, arterial and venous blood concentrations are assumed to have equilibrated [29].

Time–activity curves were fitted to the three-compartmental (two tissue) tracer kinetic model described by Hawkins et al. [30], which consists of a vascular (blood) compartment, a fluid extravascular bone tissue compartment, and a compartment representing fluoride binding into the bone matrix. Data-fitting using the nonlinear regression (NLR) method was used to estimate rate constants K_1_, k_2_, and k_3_. This was performed on a ROI basis using COMKAT software and voxelwise using PMOD software to produce parametric maps. Estimates of blood volume, partial volume effects, and dispersion were included in the model. The rate of total plasma clearance, K_i_^NLR^, was determined from calculated rate constants using the equation:

$$ {{\mathrm{K}}_{\mathrm{i}}}^{\mathrm{NLR}}={\mathrm{K}}_1\times {\mathrm{k}}_3/\left({\mathrm{k}}_2+{\mathrm{k}}_3\right) $$

For comparison, K_i_ values were also obtained using the graphical PATLAK method (K_i_^PAT^) [31].

### Bone segmentations and regions of interest (ROIs)

Cortical bone was defined as the long bone of the femur and tibia 6–8 cm from the centre of the joint space, excluding sites of tendon insertion. Trabecular bone ROIs in the proximal tibia and femoral condyle of the knee joint were drawn for both legs, maintaining a distance of 3 mm from the subchondral bone. Subchondral bone of the femur was further segmented into five regions: trochlea, medial and lateral central and posterior regions.

In addition, we identified focal areas with abnormally high increases in post-exercise uptake. These subchondral bone ROIs consisted of four or more adjacent voxels with an absolute SUV increase after exercise greater than two standard deviations above the mean SUV increase. Abnormal focal ROIs were termed ROI_focal_ and were applied to pre- and post-exercise SUV and kinetic parameter maps for analysis.

### Statistical analysis

A single mixed effects model was created to perform three analyses on the measured parameters SUV, SUVmax, K_i_^NLR^, K_1_, extraction fraction (k_3_/(k_2_ + k_3_)), k_2_, k_3_, and blood volume using general linear regression, a Laplacian fit method and with subjects set as a random effect. The first analysis tested for a significant effect of exercise on the parameters of interest (fixed effect = pre- or post-exercise scan, dependant variable = mean ROI values for given parameter). The second incorporated an ANOVA test of regression coefficients for significant differences in the response to exercise between cortical, subchondral, and trabecular bone tissues and between the step-up/drop–land legs (fixed effects = tissue type and leg, dependant variable = change in parameter ROI values after exercise). The third analysis also included an ANOVA test for regional differences in response to exercise between subchondral ROIs and step-up vs drop–land leg (fixed effects = subchondral ROI and leg, dependant variable = change in subchondral ROI values after exercise). Reported *p*-values were corrected for eight comparisons (number of parametric values tested) using a Bonferroni correction. Correlations between SUV, K_i_^NLR^, and K_i_^Pat^ were analysed using least products linear regression with a Pearson’s adjusted *R*^2^ value to evaluate goodness of fit. Image co-registration, ROI analysis, calculations, and statistical analysis were performed with software created in MATLAB 2016b (MathWorks, Natick, MA, USA).

## Results

**Fig. 2:**
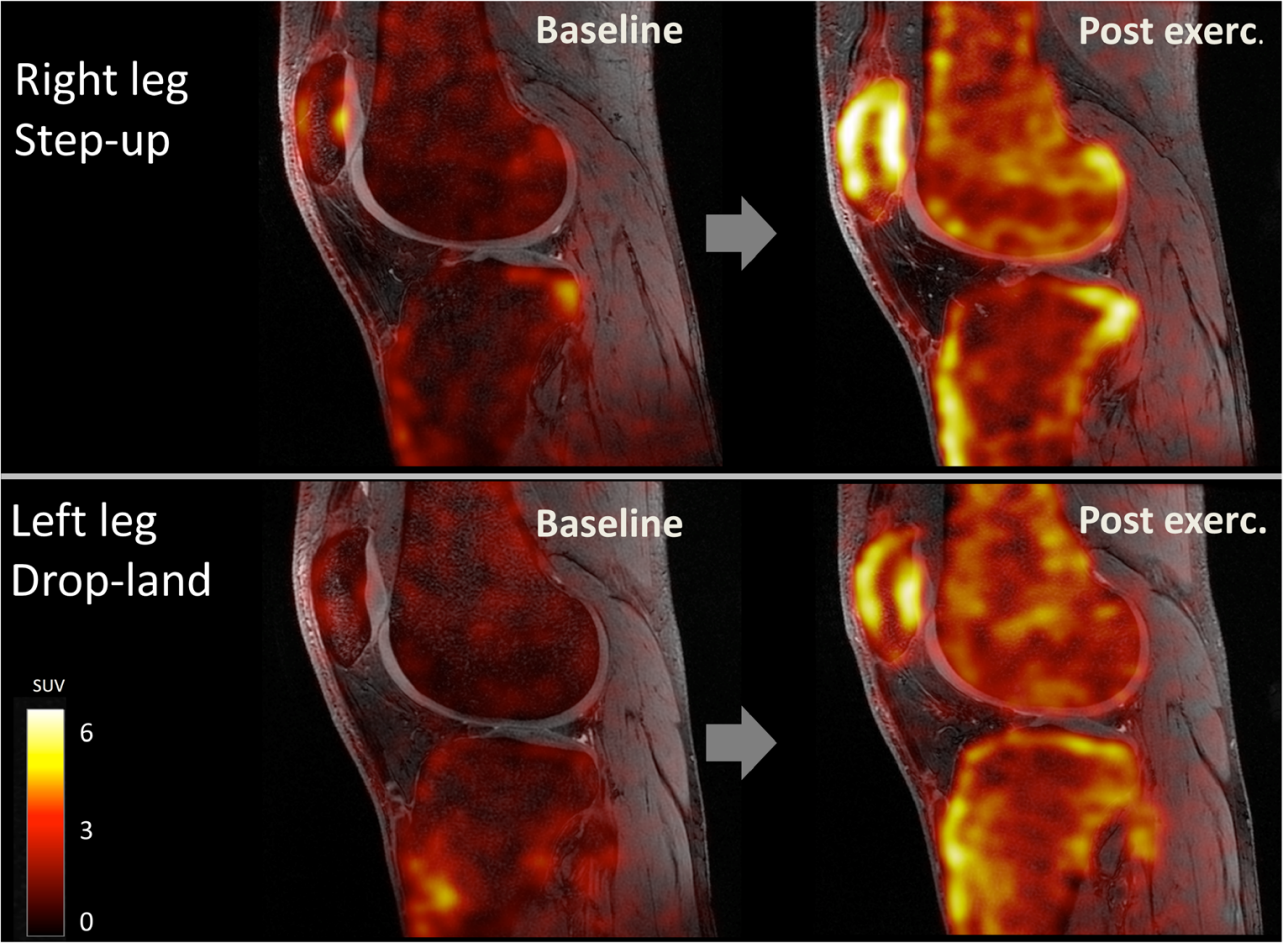
[^18^F]NaF SUV images for one subject at baseline and post exercise. Example images from each leg of one subject before and after exercise. SUV values increased significantly in all subjects after performing the one leg step-up (right leg) and one leg drop–land (left leg) exercise. SUV increases were higher in the step-up leg and varied regionally throughout the bone tissues of the knee (*p* < 0.05)

**Fig. 3:**
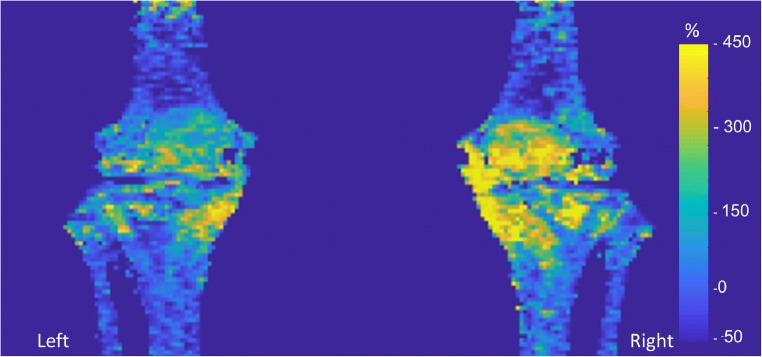
Percentage increase in NaF uptake in the step-up (*right*) leg and drop–land (*left*) leg after exercise in one subject. Mean value projection image of SUV changes in subchondral and cortical bone for one subject. Spatial distributions for a given subject, such as the higher medial versus lateral difference in this subject, were evident and varied greatly between individuals. On a group level, SUV values were higher in the right step-up leg and regional differences in subchondral ROIs were significant within each knee (*p* < 0.05)

**Fig. 4:**
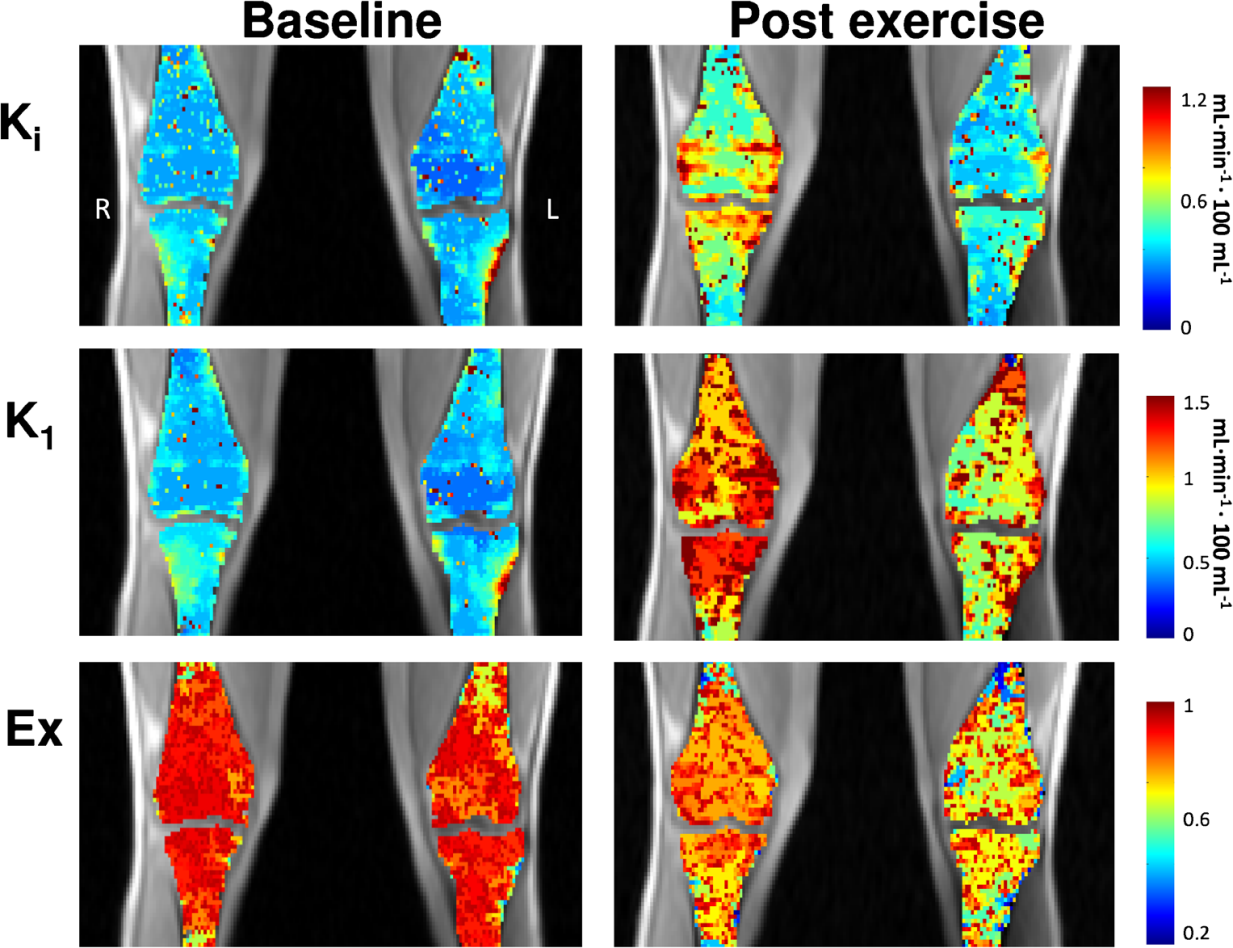
Changes in kinetic parameters after exercise in one subject. After exercise, an increase in measured K_i_, K_1_, and k_2_ values and a decrease in k_3_ values were obtained using NLR fitting. The opposing changes in k_2_ and k_3_ values both contribute to a decrease in extraction fraction (Ex) from near total extraction at baseline (k_3_/(k_2_+k_3_) ≈1) to a range of 0.35–0.8. Above are parametric maps for one subject at baseline and after exercise. Individuals had diverse patterns of activation including visible asymmetry between the step-up and drop–land leg in the post-exercise parametric maps. In subchondral bone, changes in SUV and values of all kinetic parameters, except blood volume and k_2_, varied significantly between the subregional ROIs

Exercise induced a response in [^18^F]NaF kinetics in all bone tissues (Fig. 2 and Fig. 3), with increases in SUV, SUVmax, K_i_, blood volume, and k_2_, along with a decrease in extraction fraction in all bone tissues (*p* <  0.001). In cortical bone, k_3_ increased after exercise as opposed to a post-exercise decrease of k_3_ in trabecular and subchondral bone (*p* < 0.01). SUV increases were lowest in cortical bone (47%) and highest in subchondral bone (131%) (*p* < 0.001). SUV increases were significantly higher in the step-up (right) knee (*p* < 0.05). Absolute and relative changes in SUV values and kinetic parameters for cortical, subchondral, and trabecular bone are presented in Table 1. On an individual level, regional variations in SUV increases (Fig. 3) and changes in kinetic parameters (Fig. 4) were observed between medial–lateral compartments as well as between step-up (right) and drop–land (left) legs.

**Table 1:** Absolute and relative post-exercise increases in parametric values

	Cortical	Subchondral	Trabecular
SUV *^†^	0.38 ± 0.3 (47%)	0.83 ± 0.4 (131%)	0.6 ± 0.3 (117%)
SUV_max_*	1.1 ± 0.9 (46%)	1.8 ± 0.7 (121%)	1.1 ± 0.5 (103%)
K_i_^NLR^* ml/min/100 ml	0.15 ± 0.2 (17%)	0.6 ± 0.3 (92%)	0.46 ± 0.2 (78%)
K_1_* (perfusion) ml/min/100 ml	1.61 ± 1.3 (86%)	1.38 ± 0.8 (176%)	1.2 ± 0.9 (180%)
k_2_*	0.4 ± 0.3 (264%)	0.14 ± 0.24 (533%)	0.21 ± 0.26 (604%)
k_3_*	0.16 ± 0.21 (102%)	−0.21 ± 0.22 (−23%)	−0.13 ± 0.20 (−23%)
$\frac{{\boldsymbol{k}}_{\mathbf{3}}}{{\boldsymbol{k}}_{\mathbf{3}}+{\boldsymbol{k}}_{\mathbf{2}}}$* (extraction fraction)	−0.18 ± 0.16 (−34%)	−0.23 ± 0.18 (−25%)	−0.25 ± 0.24 (−28%)
Blood* (%vol)	1.3 ± 0.39 (317%)	1.0 ± 0.36 (1051%)	0.96 ± 0.37 (981%)

Mean (± standard deviation) absolute change in SUV, and kinetic parameters for cortical, subchondral, and trabecular bone tissues of the knee. Relative increases in parentheses are percentage increase from baseline value. * Denotes significant differences in absolute post-exercise change between tissues types are marked, while † denotes significant difference in tissue response between step-up and drop–land leg (*p* < 0.05 ). In these tissues, K1 has been found to be equivalent to perfusion [28]. All pre- and post-exercise measures for all parameters are presented in supplementary material.

**Fig. 5:**
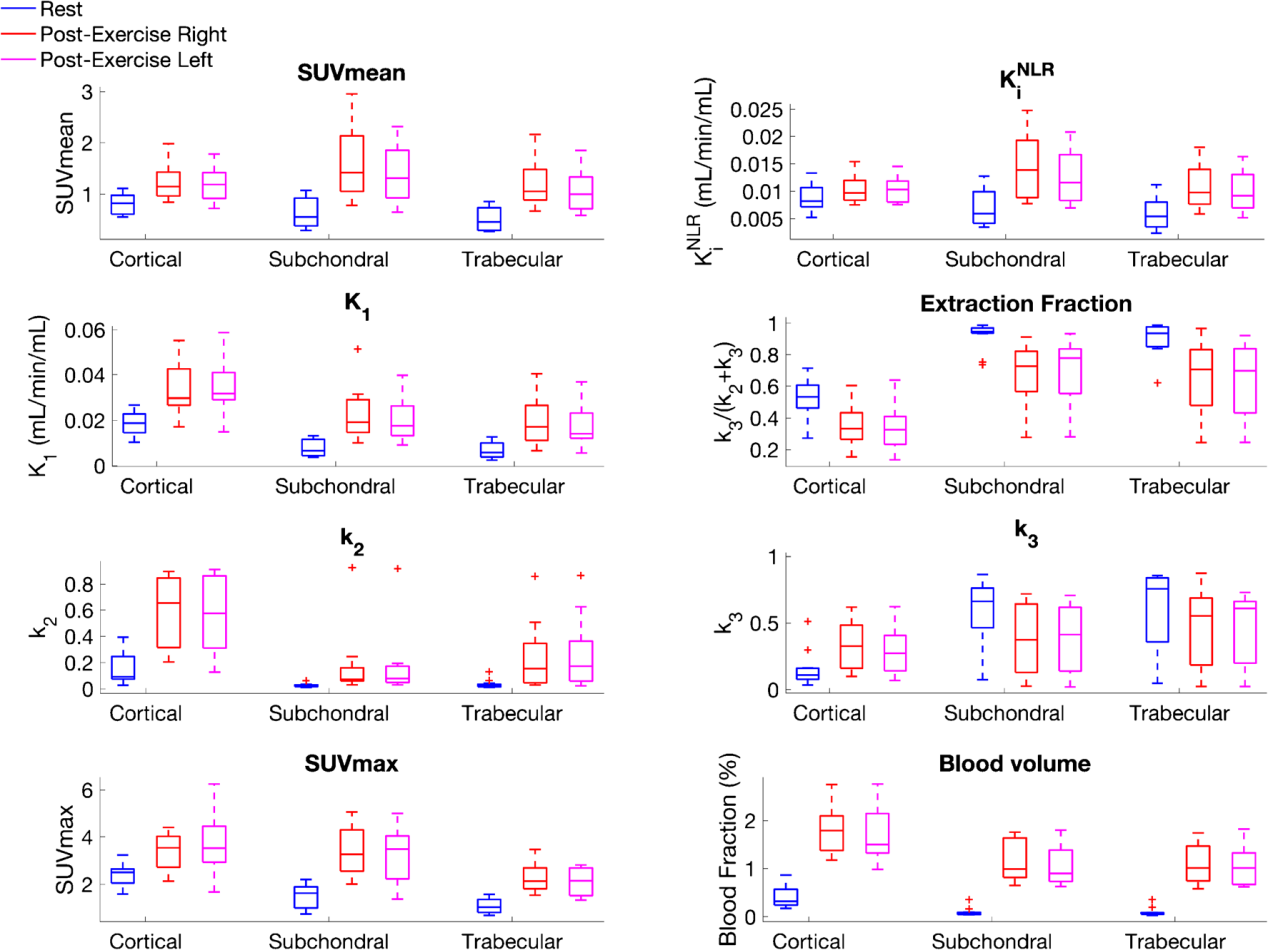
Parametric values at rest and after step-up drop–land exercise. SUV, K_i_, K_1_, k_2_, and blood volume increased significantly in all three bone tissues, in both legs, after exercise (p < 0.01). In cortical bone,l k_3_ increased while k_3_ decreased in subchondral and trabecular bone (*p* < 0.01). In all tissues, extraction fraction (k_3_/(k_2_+k_3_)) decreased significantly (*p* < 0.01) due to k_2_ changes being proportionally larger than k_3_. Outliers are presented as ’*+*’

**Fig. 6:**
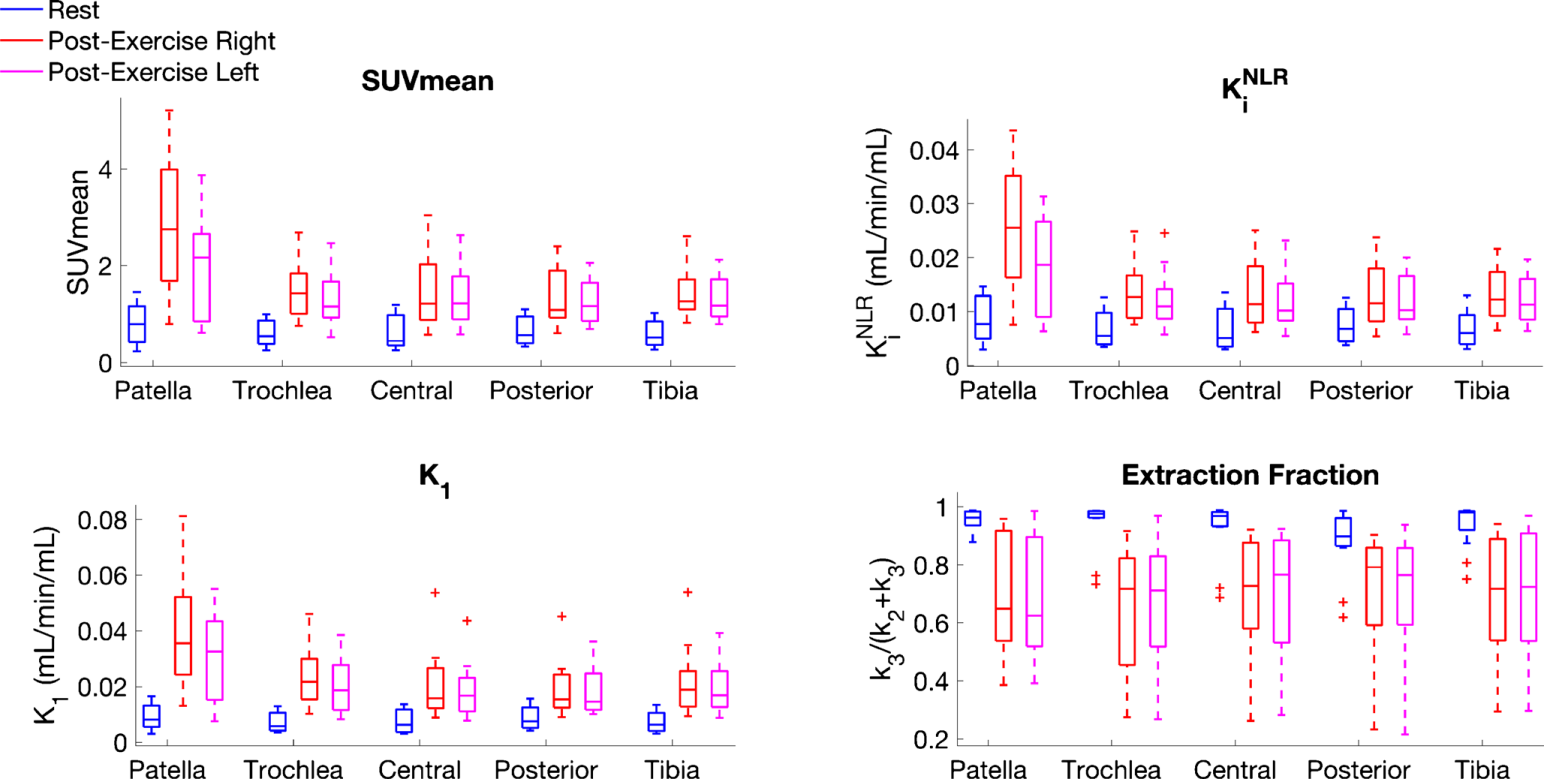
Distribution of sub-regional changes in subchondral bone [^18^F]NaF uptake parameters. The patella region of subchondral bone had larger increases in SUV (*p* < 0.01) and K_i_ (*p* < 0.01) than the mean increases in subchondral bone. The patella also had significant differences in SUV and K_i_ between step-up (right) and drop–land (left) legs (*p* < 0.05). All *p*-values are corrected for 20 comparisons using a Bonferroni correction. Outliers are presented as ’*+*’

However, on a group level, significant differences in increases were only observed in SUV, K_i_, K_1_, and blood volume of subchondral bone between the step-up leg (*p* < 0.05) and the drop–land leg (Fig. 5). All compartments of subchondral bone within each knee showed significant (*p* < 0.05) post-exercise changes of SUV and all kinetic parameters (Fig. 6) with the exception of blood volume and k_2_. The patella had the highest absolute changes in SUV and kinetic parameters (*p* < 0.05) of all the subchondral ROIs, as well as the largest differences between the step-up and drop–land legs (Fig. 6).

**Fig. 7:**
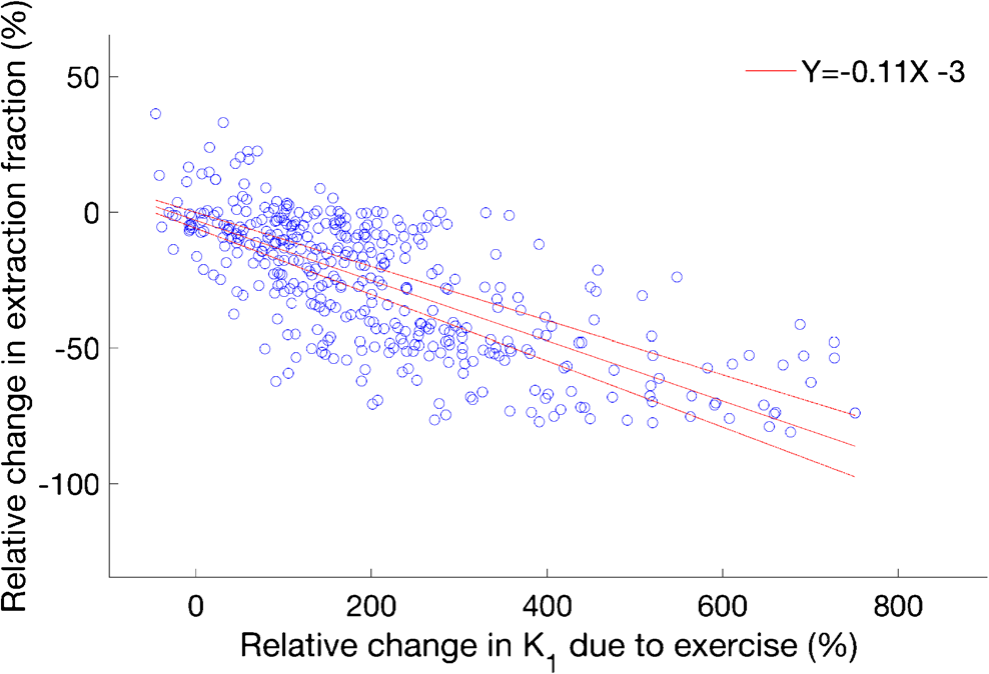
Relationship between the change in K_1_ and the change in extraction fraction. An inverse relationship was observed between the change in bone perfusion (K_1_) and the change in extraction fraction after exercise (ΔExtraction fraction= −12 × ΔK_1_ − 0, *R*^2^=0.43, *p* <0.01)

Higher post-exercise K_i_^NLR^ values were measured in all tissues. This was driven by increases in K_1_ values that exceeded the decreases in extraction fraction. An increase in fluoride flux back to blood plasma (k_2_) in all tissues contributed to a decrease of overall extraction fraction (k_3_/(k_2_ + k_3_)). The decrease in extraction fraction was found to be inversely proportional to K_1_ increases (*R*^2^ = 0.43, *p* < 0.01) (Fig. 7).

**Fig. 8:**
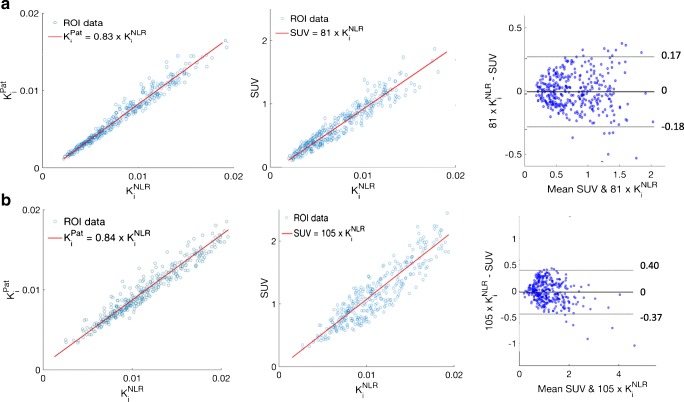
Relationship between K_i_^NLR^ and K_i_^pat^ as well as K_i_ and SUV **a** before and **b** after acute loading. K_i_NLR and Kipat maintained a similar correlation before and after exercise. However, the correlation between K_i_ and SUV changed significantly (*p* < 0.01). The slope for the linear correlation between K_i_NLR and SUV increased from 81 (**a**) to 105 (**b**) with little change in goodness of fit (*R*^2^ = 0.84 and 0.82 respectively)

K_i_ and SUV values were highly correlated (*R*^2^ > 0.8) both before and after exercise, although the correlation coefficient was altered due to a relatively larger increase in SUV values than K_i_^NLR^ values. Prior to exercise, the regression slope of K_i_^NLR^ against SUV was 85, whereas after exercise the slope increased to 105 (Fig. 7). The correlation between K_i_^NLR^ and K_i_^pat^ remained unchanged in both conditions (Fig. 8).

**Fig. 9:**
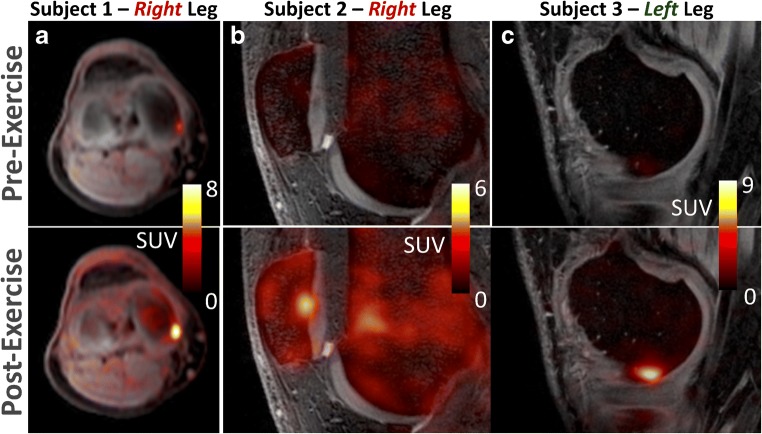
Examples of high uptake ROI_afp_ post-exercise. In six of the 12 healthy subjects, a total of eight focal points of high uptake were identified in post-exercise [18F]NaF PET SUV images such as the three examples given. The images represent cases of the focal region being visible prior to exercise and **a** maintaining a similar ratio to surrounding tissue after exercise as well as cases where the focal point was unremarkable prior to exercise (**b**), or barely discernible (**c**).

Increases in [^18^F]NaF uptake parameters did not always follow a regional or compartmental pattern. Eight ROI_focal_ were identified in the post-exercise [^18^F]NaF PET images of six subjects (Fig. 9). Of these, three of the focal points already had significantly higher activity (Fig. 9c) than the surrounding subchondral tissue at baseline, and five were unremarkable at baseline (Fig. 8a, b). The relative mean increase in SUV was 342% (209–518%) for the focal points appearing normal at baseline, and 111% (82–180%) for the focal points which were identifiable at baseline .

Differences between venous blood samples and IDIF values measured at 50 min were on average within 0.2 KBq/ml, which corresponds to a coefficient of variation (CV) of 12%. Comparing the computed IDIF between repeated acquisitions across 12 subjects, a CV of 8.4% was observed at 2 min after injection, 10.6% at 10 min, and 9.9% at 25 min. The input function of the second injection was on average 0.25 KBq/ml higher than the first injection throughout the 50-min time period across all subjects.

## Discussion

In this study, we evaluated kinetic modeling of dynamic [^18^F]NaF PET uptake to detect changes in bone physiology after an acute exercise bout. In healthy volunteers, exercise induced a significant change in uptake parameters, suggesting that [^18^F]NaF PET may provide a sensitive measure of the metabolic and hemodynamic response of bone to acute joint loading. Notably, the difference in exercise task performed by the right and left leg (step-up vs drop–land) was reflected in quantifiable differences of the patellar acute response between the two knees. Other regional differences were observed in parameter changes throughout the subchondral bone of each knee, suggesting discrepancies in the mechanical loading of these subsections. Further, focal areas of elevated [^18^F]NaF PET uptake may identify regions where there is an inconsistent response of the bone–cartilage unit to loading in areas that appear normal on MRI. Some focal points were evident prior to exercise and maintained a similar elevated uptake compared to surrounding tissue post exercise, and others were not evident until post exercise. These findings suggest that kinetic modeling of [^18^F]NaF PET may provide a promising non-invasive method to study the effects of acute loading on bone remodeling, which may increase our understanding of the effects of loading on bone and joints in healthy individuals as well as in patients with arthritis/arthrosis.

The changes in kinetic parameters observed included large increases in perfusion, as measured by fluoride influx (K_1_), and blood volume which coincided with a decrease in the rate of fluoride extraction k_3_/(k_3_ + k_2_) (Table 1 and supplementary material). This combination is indicative of a hyperemia effect, where an increased delivery of substrates exceeds consumption during a vascular response to stimulation. Changes of blood volume from near zero at baseline to a volume fraction of 1–2% after exercise indicate a vasodilation of the capillary bed as well. In humans, blood flow increases of 130% at the mid femur have been reported for bone, bone marrow included [19], as have increases in blood volume in the tibia [32] during exercise. Further, the exercise-induced hyperemia in bone marrow has been shown to be in part regulated by opposing vasodilators (nitric oxide, prostaglandin, and adenosine) and vasoconstricting factors such as the α-adrenoreceptor. [20, 33]. Although clinical bone-loading studies using [^18^F]NaF are sparse, our findings are similar to previous animal studies. Rat forelimbs were found to have increases in the rates of fluoride influx (K_1_ by 113%), fluoride metabolism (K_i_ by 133%), and fluoride incorporation into bone (k_3_ by 13%), as well as an increase in bone blood flow (26%) in cortical bone after heavy loading [24]. Discrepancies in the magnitude of change reported previously and this study may be due to differences in type and magnitude of loading, timing of the post-exercise scanning, and/or differences in bone physiology between rats and humans. Another study of [^18^F]NaF SUV values in the shaft of the rat forelimb reported an increase of 50% immediately after bone loading with comparable force magnitudes, which is similar to the 47% increase we measured in cortical bone [21]. However, the same study also observed increases in SUV to be greater with higher loading forces and that SUV values continued to increase over time peaking 4–7 days after loading. Time delays of up to 5 days have been registered between a single period of mechanical loading in vivo and observed increases of collagen and mineral apposition on surface of the loaded bone matrix [7]. Similarly, increased blood flow in the rat ulna has been found to peak 5 days after loading [22]. It should be noted that the aforementioned studies used bone-loading devices as opposed to the more physiologic type of exercise in the present study. Such loading studies also tend to employ loads that exceed forces under normal exercise, which means the time and load dependency of a measured response in bone requires more study. Still, the interconnection of vascular fluctuations in bone with an anabolic effect on bone remodelling has been established [34–36].

The changes in [^18^F]NaF kinetics, total uptake, and SUV induced by exercise in this study suggest a localised response in bone tissue that may be load-dependant, with high inter-subject variability. Although the step-up and jump–landing exercises are functionally different, they result in a similar loading environment in the knee joint. The tibiofemoral joint experiences compressive forces from both ground reaction forces and muscle forces, while the patella experiences tensile forces from the quadriceps and compressive forces through its articulation with the trochlea. Stair ascent and descent are similar biomechanical tasks to the step-up and jump–landing exercises. There are slight differences in peak ground reaction forces between stair ascent:(1.1 bodyweights) versus stair descent (1.4 bodyweights), but both involve peak quadriceps forces of 2.4 bodyweights [37]. As a result, the right and left tibiofemoral joints may have experienced similar loading, but the right patella may have transmitted greater quadriceps forces than the left. In this study, both legs showed the highest SUV and K_i_^NLR^ increases in the patella, which may correspond with the large forces transmitted through the patella. The right patella, however, demonstrated higher SUV and K_i_^NLR^ than the left, which may be explained by the greater quadriceps forces in the step-up activity. The whole-body biomechanics of the two tasks are similar, but tissue-level strains have high inter-subject and spatiotemporal variability [37]. Differences in movement dynamics, varus/valgus alignment, muscle strength, and coordination strategies could all contribute to a high degree of inter-subject variability in forces applied on the bones. Even if the applied forces were identical between subjects, local bone strain is also dependent on the geometry and microstructure of each individual’s bones, resulting in further variability. We also observed intersubject discrepancies in the spatial distribution of kinetic/SUV changes, which may reflect inter-subject differences in bone strain. Furthermore, these discrepancies imply that the physiological mechanisms responsible for the altered kinetics are locally regulated, as opposed to a uniform increase in flow to the bones of the knee region.

SUV values increased more than K_i_ values even though the two were correlated before and after exercise. One reason for this bias could be the 0.25 KBq/ml extra blood activity (an increase of approx 10% during the 30 to 50 min time period) after the second injection. SUV may also be artificially elevated due to a reduction in the extraction of fluoride in other areas of the body, such as the kidneys. Exercise is known to reduce blood flow to other areas of the body, including the kidneys, via activation of the sympathetic nerve system [38]. Increasing the proportion of fluoride available to bone tissue in the knee would not alter K_i_, as it is accounted for by the input function. SUV, on the other hand, is calibrated to the injected dose and does not account for remaining activity in the blood from the first injection or for changes in fluoride extraction elsewhere in the body including the kidneys [38].

Findings of focal areas in subchondral bone with abnormally high increases in ^18^F-NaF uptake after exercise, ROI_focal_, are of particular interest. Many of these regions showed no abnormalities in ^18^F-NaF uptake compared to adjacent regions at baseline pre-exercise or structural subchondral bone abnormalities on MRI. This suggests that ^18^F-NaF PET may identify regions where there is an improper response of the bone–cartilage unit to acute loading in areas that appear structurally normal on MRI. Biomarkers that can effectively assess early breakdown of joint function, before structural changes are seen, are crucial for the development and evaluation of disease-modifying treatments, and increased ^18^F-NaF uptake detected after acute loading may be a promising candidate. Longitudinal studies are necessary to identify the relationship of these regions of increased uptake after acute joint stress and the pathophysiology of bone and cartilage degeneration in these regions.

There are several limitations in this study. First, the study was designed for high loading of the knee joint to ensure detection of an eventual osteogenic response from exercise. The study design is, for this reason, limited in its ability to interpret the relationship between the amount of stress applied to bone tissue and the corresponding response. The exercise paradigm included both high strain magnitudes and high strain rates on the knee joint [7], and used the maximum number of cycles reported to ensure saturation of mechanostimulation [5]. To determine the relationship between the stress applied to bone and the resulting response, all three of these elements would have to be varied in a controlled fashion.

This study used a cross-sectional analysis of healthy volunteers with no symptoms or history of knee injury or pain. Further, no histopathology was acquired in ROIs with abnormal focal increases in uptake after exercise. While increased bone activity detected by ^18^F-NaF after acute loading is suggestive of higher bone loading due to a breakdown of proper joint function, longitudinal studies or histopathology are needed to confirm degenerative changes in these areas. A study focusing on patient groups with known pathology would help assess the utility of stimulating bone tissue in diagnostics. The number of subjects in this study is also small, which limits its ability to identify factors such as BMI, varus/valgus alignment, disease, or activity level that could alter the kinetics in bone tissue. The exercise in this study is also inappropriate for many patient groups. A paradigm designed for patients could, however, induce a similar response, given that ~95% of the osteogenic effect can be achieved after climbing only ~20 to 40 steps (~2000 μƐ in compression) [5, 39, 40].

Furthermore, there are technical limitations to consider. Kinetic studies are more complicated than conventional imaging, requiring more computation, expertise, and the robust determination of an input function. The IDIF methods used in this study have previously been shown to correlate with late-stage venous blood measures and to be reproducible. Further, input function values in this study correspond well with literature values for arterial sampling [29], but using arterial sampling is still considered a gold standard. The use of two consecutive injections was accounted for, but may create an adverse bias in the results, as blood activity levels from the second injection were slightly higher than the first. The blood volume measured by this method is difficult to verify independently in humans with a gold standard. Lastly, despite the numerous advantages from combining PET imaging with MRI in knee examinations, PET/MR is also known to underestimate SUV values by approximately 10% due to MR-based attenuation correction of its PET images [41].

The ability to image the physiological response of bone tissue to loading would be a valuable clinical tool. Decreased mechanosensitivity in bone tissue has been identified in osteoporosis [42, 43] and patients with spinal cord injury [32], and to decline in the general population with age [44–46]. Medicinal treatment or therapies employing mechanostimulation including exercise [47], muscle stimulation [6, 48], vibration therapy [49], or drug treatment [17, 50] could better be imaged using [^18^F]NaF PET. For prostate and breast cancer, studies have shown bone-loading exercise to be a safe and viable suppressant of tumor growth in bone metastasis [51, 52]. The use of kinetics, even at rest, haa been proven to be clinically feasible and has the potential to offer more information. For instance, K_i_ has been shown to be highly reproducible in the human spine [53, 54] and to be more valuable in monitoring osteoporosis patients than SUV values [55]. Subchondral bone changes that are present prior to and during the development of OA have demonstrated increased bone blood flow and bone remodeling in [^18^F]NaF PET studies [15]. An association between pain and mean normalized standardized uptake values (SUV) has been established in different bone types [12, 15, 16], and increased [^18^F]NaF SUV values may precede visible lesions [12, 14]. Thus, detecting early changes in bone remodeling and bone physiology with [^18^F]NaF kinetics could help in understanding disease. In those cases where pathology causing elevated SUV is poorly understood or where there is weak contrast to healthy bone, the response of bone tissue to mechanostimulation could further elucidate dysfunction.

## Conclusions

Loading acutely alters bone physiology affecting [^18^F]NaF-PET kinetics, with a local response depending on tissue, site, and exercise. Our data support previous evidence of mechanostimulation initiating an early hyperemia phase in bone adaptation. These findings suggest that kinetic modeling of [^18^F]NaF PET may provide a non-invasive method to study the effects of acute loading on joint biomechanics and bone remodeling, which may have large implications for our understanding of early stages of arthritis/arthrosis.

## Electronic supplementary material

ESM 1 (PDF 78 kb)

## References

[1] Pasco JA, Mohebbi M, Holloway KL, Brennan-Olsen SL, Hyde NK, Kotowicz MA (2017). Musculoskeletal decline and mortality: prospective data from the Geelong Osteoporosis Study. J Cachexia Sarcopenia Muscle.

[2] Ray R, Clement ND, Aitken SA, McQueen MM, Court-Brown CM, Ralston SH (2017). High mortality in younger patients with major osteoporotic fractures. Osteoporos Int.

[3] Milte R, Crotty M (2014). Musculoskeletal health, frailty and functional decline. Best Pract Res Clin Rheumatol.

[4] Bliuc D, Alarkawi D, Nguyen TV, Eisman JA, Center JR (2015). Risk of subsequent fractures and mortality in elderly women and men with fragility fractures with and without osteoporotic bone density: the Dubbo Osteoporosis Epidemiology Study. J Bone Miner Res.

[5] Burr DB, Robling AG, Turner CH (2002). Effects of biomechanical stress on bones in animals. Bone..

[6] Hart NH, Nimphius S, Rantalainen T, Ireland A, Siafarikas A, Newton RU (2017). Mechanical basis of bone strength: influence of bone material, bone structure and muscle action. J Musculoskelet Neuronal Interact.

[7] Rosa N, Simoes R, Magalhães FD, Marques AT (2015). From mechanical stimulus to bone formation: a review. Med Eng Phys.

[8] Weatherholt AM, Fuchs RK, Warden SJ (2013). Cortical and trabecular bone adaptation to incremental load magnitudes using the mouse tibial axial compression loading model. Bone..

[9] Kaji H (2014). Interaction between muscle and bone. J Bone Metab.

[10] Tyrovola JB (2015). The “mechanostat theory” of frost and the OPG/RANKL/RANK system. J Cell Biochem.

[11] Klein-Nulend J, Bakker AD, Bacabac RG, Vatsa A, Weinbaum S (2013). Mechanosensation and transduction in osteocytes. Bone..

[12] Savic D, Pedoia V, Seo Y, Yang J, Bucknor M, Franc BL (2016). Imaging bone-cartilage interactions in osteoarthritis using [(18)F]-NaF PET-MRI. Mol Imaging.

[13] Kogan F, Fan AP, Gold GE (2016). Potential of PET-MRI for imaging of non-oncologic musculoskeletal disease. Quant Imaging Med Surg.

[14] Kogan F, Fan AP, McWalter EJ, Oei EHG, Quon A, Gold GE (2017). PET/MRI of metabolic activity in osteoarthritis: a feasibility study. J Magn Reson Imaging.

[15] Draper CE, Quon A, Fredericson M, Besier TF, Delp SL, Beaupre GS (2012). Comparison of MRI and ^18^F-NaF PET/CT in patients with patellofemoral pain. J Magn Reson Imaging.

[16] Kobayashi N, Inaba Y, Tateishi U, Ike H, Kubota S, Inoue T (2015). Comparison of 18F-fluoride positron emission tomography and magnetic resonance imaging in evaluating early-stage osteoarthritis of the hip. Nucl Med Commun.

[17] Blake GM, Siddique M, Frost ML, Moore AEB, Fogelman I (2014). Imaging of site specific bone turnover in osteoporosis using positron emission tomography. Curr Osteoporos Rep.

[18] Heinonen I, Kemppainen J, Fujimoto T, Knuuti J, Kalliokoski KK (2019). Increase of glucose uptake in human bone marrow with increasing exercise intensity. Int J Sport Nutr Exerc Metab.

[19] Heinonen I, Kemppainen J, Kaskinoro K, Langberg H, Knuuti J, Boushel R (2013). Bone blood flow and metabolism in humans: effect of muscular exercise and other physiological perturbations. J Bone Miner Res.

[20] Heinonen I, Boushel R, Hellsten Y, Kalliokoski K (2018). Regulation of bone blood flow in humans: the role of nitric oxide, prostaglandins, and adenosine. Scand J Med Sci Sports.

[21] Silva MJ, Uthgenannt BA, Rutlin JR, Wohl GR, Lewis JS, Welch MJ (2006). In vivo skeletal imaging of 18F-fluoride with positron emission tomography reveals damage- and time-dependent responses to fatigue loading in the rat ulna. Bone..

[22] Muir P, Sample SJ, Barrett JG, McCarthy J, Vanderby R, Markel MD (2007). Effect of fatigue loading and associated matrix microdamage on bone blood flow and interstitial fluid flow. Bone..

[23] De Souza RL, Matsuura M, Eckstein F, Rawlinson SCF, Lanyon LE, Pitsillides AA (2005). Non-invasive axial loading of mouse tibiae increases cortical bone formation and modifies trabecular organization: a new model to study cortical and cancellous compartments in a single loaded element. Bone..

[24] Tomlinson RE, Silva MJ, Shoghi KI (2012). Quantification of skeletal blood flow and fluoride metabolism in rats using PET in a pre-clinical stress fracture model. Mol Imaging Biol.

[25] Turner CH (1998). Three rules for bone adaptation to mechanical stimuli. Bone..

[26] Kogan F, Levine E, Chaudhari AS, Monu UD, Epperson K, Oei EHG (2018). Simultaneous bilateral-knee MR imaging. Magn Reson Med.

[27] Wagenknecht G, Kaiser H-J, Mottaghy FM, Herzog H (2013). MRI for attenuation correction in PET: methods and challenges. MAGMA..

[28] Haddock B, Fan AP, Jørgensen NR, Suetta C, Gold GE, Kogan F. Kinetic [18F]-fluoride of the knee in Normal volunteers. Clin Nucl Med. 2019;44(5):377–85. 10.1097/RLU.0000000000002533 PMC6449188 30888996

[29] Blake GM, Siddique M, Puri T, Frost ML, Moore AE, Cook GJR (2012). A semipopulation input function for quantifying static and dynamic 18F-fluoride PET scans. Nucl Med Commun.

[30] Hawkins RA, Choi Y, Huang SC, Hoh CK, Dahlbom M, Schiepers C (1992). Evaluation of the skeletal kinetics of fluorine-18-fluoride ion with PET. J Nucl Med.

[31] Patlak CS, Blasberg RG, Fenstermacher JD (1983). Graphical evaluation of blood-to-brain transfer constants from multiple-time uptake data. J Cereb Blood Flow Metab.

[32] Draghici AE, Potart D, Hollmann JL, Pera V, Fang Q, DiMarzio CA (2018). Near infrared spectroscopy for measuring changes in bone hemoglobin content after exercise in individuals with spinal cord injury. J Orthop Res.

[33] Heinonen I, Wendelin-Saarenhovi M, Kaskinoro K, Knuuti J, Scheinin M, Kalliokoski KK (2013). Inhibition of α-adrenergic tone disturbs the distribution of blood flow in the exercising human limb. Am J Physiol Heart Circ Physiol.

[34] McCarthy I (2006). The physiology of bone blood flow: a review. J Bone Joint Surg Am.

[35] Knothe Tate ML, Steck R, Forwood MR, Niederer P (2000). In vivo demonstration of load-induced fluid flow in the rat tibia and its potential implications for processes associated with functional adaptation. J Exp Biol.

[36] Tami AE, Nasser P, Verborgt O, Schaffler MB, Knothe Tate ML (2002). The role of interstitial fluid flow in the remodeling response to fatigue loading. J Bone Miner Res.

[37] Martelli S, Pivonka P, Ebeling PR (2014). Femoral shaft strains during daily activities: implications for atypical femoral fractures. Clin Biomech (Bristol, Avon).

[38] Haddock BT, Francis ST, Larsson HBW, Andersen UB (2018). Assessment of perfusion and oxygenation of the human renal cortex and medulla by quantitative MRI during handgrip exercise. J Am Soc Nephrol.

[39] Wu Q, Sample SJ, Baker TA, Thomas CF, Behan M, Muir P (2009). Mechanical loading of a long bone induces plasticity in sensory input to the central nervous system. Neurosci Lett.

[40] Umemura Y, Ishiko T, Yamauchi T, Kurono M, Mashiko S (1997). Five jumps per day increase bone mass and breaking force in rats. J Bone Miner Res.

[41] Aznar MC, Sersar R, Saabye J, Ladefoged CN, Andersen FL, Rasmussen JH (2014). Whole-body PET/MRI: the effect of bone attenuation during MR-based attenuation correction in oncology imaging. Eur J Radiol.

[42] Klein-Nulend J, van Oers RFM, Bakker AD, Bacabac RG (2015). Bone cell mechanosensitivity, estrogen deficiency, and osteoporosis. J Biomech.

[43] Bakker AD, Klein-Nulend J, Tanck E, Heyligers IC, Albers GH, Lips P (2006). Different responsiveness to mechanical stress of bone cells from osteoporotic versus osteoarthritic donors. Osteoporos Int.

[44] Torvinen S, Kannus P, Sievänen H, Järvinen TAH, Pasanen M, Kontulainen S (2002). Effect of four-month vertical whole body vibration on performance and balance. Med Sci Sports Exerc.

[45] Ireland A, Maden-Wilkinson T, Ganse B, Degens H, Rittweger J (2014). Effects of age and starting age upon side asymmetry in the arms of veteran tennis players: a cross-sectional study. Osteoporos Int.

[46] Hemmatian H, Bakker AD, Klein-Nulend J, van Lenthe GH (2017). Aging, osteocytes, and mechanotransduction. Curr Osteoporos Rep.

[47] Barry DW, Kohrt WM (2008). Exercise and the preservation of bone health. J Cardiopulm Rehabil Prev.

[48] Qin YX, Lam H, Ferreri S, Rubin C (2010). Dynamic skeletal muscle stimulation and its potential in bone adaptation. J Musculoskelet Neuronal Interact.

[49] Thompson WR, Yen SS, Rubin J (2014). Vibration therapy: clinical applications in bone. Curr Opin Endocrinol Diabetes Obes.

[50] Frost ML, Blake GM, Cook GJR, Marsden PK, Fogelman I (2009). Differences in regional bone perfusion and turnover between lumbar spine and distal humerus: (18)F-fluoride PET study of treatment-naïve and treated postmenopausal women. Bone..

[51] Hart NH, Newton RU, Spry NA, Taaffe DR, Chambers SK, Feeney KT (2017). Can exercise suppress tumour growth in advanced prostate cancer patients with sclerotic bone metastases? A randomised, controlled study protocol examining feasibility, safety and efficacy. BMJ Open.

[52] Lynch ME, Fischbach C (2014). Biomechanical forces in the skeleton and their relevance to bone metastasis: biology and engineering considerations. Adv Drug Deliv Rev.

[53] Frost ML, Blake GM, Park-Holohan S-J, Cook GJR, Curran KM, Marsden PK (2008). Long-term precision of 18F-fluoride PET skeletal kinetic studies in the assessment of bone metabolism. J Nucl Med.

[54] Al-Beyatti Y, Siddique M, Frost ML, Fogelman I, Blake GM (2012). Precision of ^18^F-fluoride PET skeletal kinetic studies in the assessment of bone metabolism. Osteoporos Int.

[55] Blake GM, Siddique M, Frost ML, Moore AEB, Fogelman I (2012). Quantitative PET imaging using (18)F sodium fluoride in the assessment of metabolic bone diseases and the monitoring of their response to therapy. PET Clin.

