# Supplementary material for: Assessment of acute bone loading in humans using [18F]NaF PET/MRI
Source: Eur J Nucl Med Mol Imaging. 2019 Aug 5;46(12):2452–63. doi: 10.1007/s00259-019-04424-2 (PMC6813760; doi:10.1007/s00259-019-04424-2)
Supplement: Supplementary file 1 — (PDF 78 kb) [file 259_2019_4424_MOESM1_ESM.pdf]

## Parametric values for [18F]NaF kinetics of the knee at rest and after exercise

| Rest | Patella - Subchondral | Femur- Subchondral | Tibia- Subchondral | Tendon insert | Femur- Cortical (Shaft) | Tibia- Cortical (Shaft) | Femur- Trabecular | Tibia- Trabecular |
|---|---|---|---|---|---|---|---|---|
| **SUV** | 0.7 (0.43 - 1.02) | 0.52 (0.38 - 0.94) | 0.52 (0.37 - 0.85) | 0.89 (0.58 - 1.14) | 0.78 (0.55 - 1.03) | 0.84 (0.7 - 0.91) | 0.4 (0.27 - 0.79) | 0.52 (0.35 - 0.71) |
| **$SUV_{max}$** | 1.93 (1.18 - 2.59) | 2.49 (1.91 - 3.11) | 1.98 (1.35 - 2.54) | 3.0 (2.09 - 4.6) | 3.12 (2.51 - 3.62) | 2.24 (1.91 - 2.8) | 1.35 (0.85 - 1.73) | 1.4 (1.12 - 1.63) |
| **$K_i^{NLR}$** mL/min/100mL | 0.81 (0.44 - 1.09) | 0.56 (0.43 - 1.0) | 0.6 (0.4 - 0.93) | 1.07 (0.72 - 1.4) | 0.82 (0.61 - 1.1) | 0.87 (0.76 - 0.98) | 0.43 (0.27 - 0.82) | 0.58 (0.41 - 0.81) |
| **$K_1$** mL/min/100mL | 0.86 (0.52 - 1.13) | 0.66 (0.47 - 1.17) | 0.64 (0.41 - 1.04) | 1.29 (0.74 - 1.6) | 1.67 (1.22 - 2.42) | 1.88 (1.34 - 2.19) | 0.44 (0.29 - 0.94) | 0.67 (0.46 - 1.11) |
| **$k_3/(k_3+k_2)$** | 0.97 (0.91 - 0.99) | 0.95 (0.92 - 0.97) | 0.98 (0.92 - 0.99) | 0.95 (0.9 - 0.96) | 0.51 (0.43 - 0.61) | 0.48 (0.41 - 0.56) | 0.98 (0.94 - 0.99) | 0.89 (0.78 - 0.98) |
| **$K_i^{pat}$** mL/min/100mL | 0.69 (0.38 - 0.99) | 0.51 (0.33 - 0.9) | 0.5 (0.34 - 0.81) | 0.94 (0.64 - 1.31) | 0.62 (0.48 - 0.95) | 0.67 (0.58 - 0.77) | 0.38 (0.23 - 0.73) | 0.49 (0.33 - 0.63) |
| **Perf** mL/min/100mL | 0.86 (0.52 - 1.13) | 0.66 (0.47 - 1.17) | 0.64 (0.41 - 1.04) | 1.29 (0.74 - 1.6) | 1.67 (1.22 - 2.42) | 1.88 (1.34 - 2.19) | 0.44 (0.29 - 0.94) | 0.67 (0.46 - 1.11) |
| **Blood (%vol)** | 0.04 (0.03 - 0.06) | 0.07 (0.05 - 0.13) | 0.05 (0.03 - 0.07) | 0.05 (0.04 - 0.06) | 0.38 (0.22 - 0.64) | 0.35 (0.27 - 0.48) | 0.06 (0.04 - 0.08) | 0.09 (0.06 - 0.12) |

Supplementary Table 1 **Parametric values for bone tissues of the patella, tibia and femur at rest.**

*Median parametric values from different bone tissue with interquartile range in parenthesis. Measures are further divided into the tibial, femoral and patellar bones.*

| <u>Post-Exercise</u> | Patella | Femur-Subchondral | Tibia-Subchondral | Femur- Shaft | Tibia-Shaft | Femur-Trabecular | Tibia-Trabecular | Tendon insert |
|---|---|---|---|---|---|---|---|---|
| **SUV** | 2.34 (1.16 - 3.23) | 1.2 (0.96 - 1.9) | 1.2 (1.04 - 1.72) | 1.26 (0.88 - 1.42) | 1.13 (0.94 - 1.33) | 1.03 (0.79 - 1.45) | 1.03 (0.87 - 1.36) | 1.8 (1.21 - 2.54) |
| **$SUV_{max}$** | 6.11 (3.55 - 7.89) | 4.9 (3.95 - 7.82) | 4.52 (4.08 - 4.79) | 4.28 (3.59 - 5.17) | 3.21 (2.48 - 4.44) | 2.5 (2.12 - 2.95) | 2.53 (1.75 - 3.09) | 5.94 (4.49 - 10.1) |
| **$K_i^{NLR}$** mL/min/100mL | 1.83 (1.22 - 2.89) | 1.15 (0.81 - 1.67) | 1.22 (0.89 - 1.63) | 1.03 (0.92 - 1.27) | 0.99 (0.81 - 1.15) | 0.99 (0.7 - 1.36) | 0.96 (0.86 - 1.23) | 2.14 (1.43 - 2.8) |
| **$K_1$** mL/min/100mL | 2.93 (1.87 - 4.68) | 1.64 (1.26 - 2.64) | 1.77 (1.3 - 2.55) | 2.62 (2.23 - 3.71) | 3.85 (2.99 - 4.77) | 1.58 (0.9 - 2.33) | 1.71 (1.41 - 3.06) | 3.71 (2.0- 4.01) |
| **$k_3/(k_3+k_2)$** | 0.63 (0.55 - 0.9) | 0.75 (0.56 - 0.85) | 0.71 (0.53 - 0.91) | 0.4 (0.32 - 0.47) | 0.24 (0.2 - 0.37) | 0.76 (0.53 - 0.94) | 0.58 (0.38 - 0.89) | 0.7 (0.52 - 0.84) |
| **$K_i^{pat}$** mL/min/100mL | 1.63 (1.08 - 2.5) | 0.98 (0.66 - 1.39) | 0.98 (0.76 - 1.28) | 0.84 (0.73 - 1.02) | 0.77 (0.64 - 0.92) | 0.83 (0.6 - 1.11) | 0.82 (0.68 - 1) | 1.7 (1.26 - 2.19) |
| **Perf** mL/min/100mL | 2.93 (1.87 - 4.68) | 1.64 (1.26 - 2.64) | 1.77 (1.3 - 2.55) | 2.62 (2.23 - 3.71) | 3.85 (2.99 - 4.77) | 1.58 (0.9 - 2.33) | 1.71 (1.41 - 3.06) | 3.71 (2.0 - 4.01) |
| **Blood (%vol)** | 1.05 (0.72 - 1.36) | 0.94 (0.83 - 1.57) | 0.86 (0.74 - 1.38) | 1.9 (1.47 - 2.34) | 1.49 (1.2 - 1.86) | 0.91 (0.74 - 1.27) | 1.03 (0.68 - 1.44) | 1.2 (0.91 - 1.52) |

Supplementary Table 2 **Post exercise parametric values for different bone - tissue type combinations**

*Median parametric values from different bone tissue with interquartile range in parenthesis. Parameters obtained from dynamic scan started on average 8 minutes after step-up drop-land exercise.*
